# Imported Infections with *Mansonella perstans* Nematodes, Italy

**DOI:** 10.3201/eid2309.170263

**Published:** 2017-09

**Authors:** Federico Gobbi, Anna Beltrame, Dora Buonfrate, Silvia Staffolani, Monica Degani, Maria Gobbo, Andrea Angheben, Stefania Marocco, Zeno Bisoffi

**Affiliations:** Sacro Cuore Hospital, Verona, Italy (F. Gobbi, A. Beltrame, D. Buonfrate, M. Degani, M. Gobbo, A. Angheben, S. Marocco, Z. Bisoffi);; Azienda Ospedaliero Universitaria Umberto I-Lancisi-Salesi, Ancona, Italy (S. Staffolani)

**Keywords:** Mansonella perstans, parasites, nematodes, filariasis, imported infections, eosinophilia, microfilaremia, immigrants, expatriates, Italy

## Abstract

We report 74 patients in Italy infected with *Mansonella perstans* nematodes, a poorly described filarial parasite. *M. perstans* nematodes should be included in the differential diagnosis for patients with eosinophilia from disease-endemic countries. Serologic analysis is useful for screening, and testing for microfilaremia in peripheral blood should be performed for parasite-positive patients.

## Abstract 2

These infections should be included in differential diagnoses for patients with eosinophilia from disease-endemic countries.

*Mansonella perstans* is a filarial nematode present in 33 countries in sub-Saharan Africa; sporadic cases have been reported in Latin America, mostly in the Caribbean and along the Atlantic coast (*1*); ≈20% of inhabitants of disease-endemic countries are infected (*2*). Flies of the genus *Culicoides* transmit infective larvae to humans. Larvae transform into macrofilariae, which live in serous cavities of the human body, where they produce microfilariae, which are released into peripheral blood 9–12 months after infection.

Few studies/case series have reported signs and symptoms (e.g., subcutaneous edema, rash, abdominal pain, eosinophilia) caused by infection with *M. perstans* nematodes because the parasite is widespread in remote areas and infected persons usually have other parasitic infections that could contribute to clinical manifestations (*1*). Diagnosis is based on detection of microfilariae in peripheral blood (*3*). An ELISA that uses antigens of *Acanthocheilonema vitae* nematodes is available but is not specific for *Mansonella* spp.

Optimal treatment is still debated. Many drugs have been used, including diethylcarbamazine, ivermectin, mebendazole, levamisole, albendazole, and thiabendazole (*1*). Doxycycline, which is active against the endosymbiont *Wolbachia* spp., showed good efficacy in a clinical trial (*4*), but comparisons of the efficacy of this drug with other treatments are lacking. Most case series identified in countries to which *M. perstans* nematodes are not endemic have not been reported. The purpose of this study was to analyze the clinical, epidemiologic, and laboratory characteristics of patients infected with *M. perstans* nematodes who were given a diagnosis at the Center for Tropical Diseases at Sacro Cuore Hospital in Negrar, Verona, Italy.

## The Study

**Figure 1:**
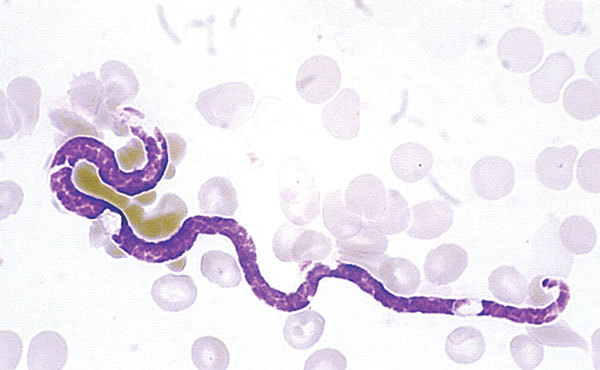
Microfilaria in a patient infected with *Mansonella perstans* nematodes, Italy. Giemsa stain, 200 µm x 4 µm, original magnification x1,000.

This retrospective study was approved by the Ethics Committee of Sacro Cuore Hospital in November 2016 (study protocol no. 56014). We reviewed medical records of patients admitted to Sacro Cuore Hospital during January 1, 1993–January 1, 2016. Inclusion criteria were available information about the most likely country of acquisition of the infection and presence of *M. perstans* microfilaremia (Figure 1).

A total of 82 patients were considered for inclusion; 8 were excluded because information was incomplete. Thus, 74 patients, 23 immigrants and 51 expatriates, were included in the analysis. Immigrants were persons who were born in disease-endemic areas and then settled in Italy. Expatriates were persons from Italy residing in disease-endemic areas. Immigrants were younger than expatriates. Mean ages were 26.8 (range 5–51) years for immigrants and 55.6 (range 12–76) years for expatriates. Most (70.3%) patients were males.

We detected microfilaremia by using a leukoconcentration method with 13-mL samples of venous blood. Microfilarial density was measured by examination of Giemsa-stained thick blood smears prepared from 100 µL of blood. We also performed retrospective ELISA for detection of filariasis (Bordier Affinity Products SA, Crissier, Switzerland) on available serum samples.

When necessary, we conducted other investigations to exclude other parasitic infections or other causes of eosinophilia. Other helminth infections were diagnosed by microscopic examination of multiple stool samples; agar stool culture (for hookworm and *Strongyloides stercoralis*); skin-snip (for *Onchocerca volvulus*); and serologic analysis (in-house immunofluorescence test for *S. stercoralis*; commercial immunofluorescence test until 2012, and an ELISA after 2012 for *Schistosoma* spp).

For each patient, information on clinical history, country of exposure, laboratory examinations, and treatment was obtained from medical records and entered into a study-specific database (Epi Info version 3.5.1; Centers for Disease Control and Prevention, Atlanta, GA, USA). Qualitative data were reported as frequencies and percentages, and quantitative data as medians and interquartile ranges.

**Figure 2:**
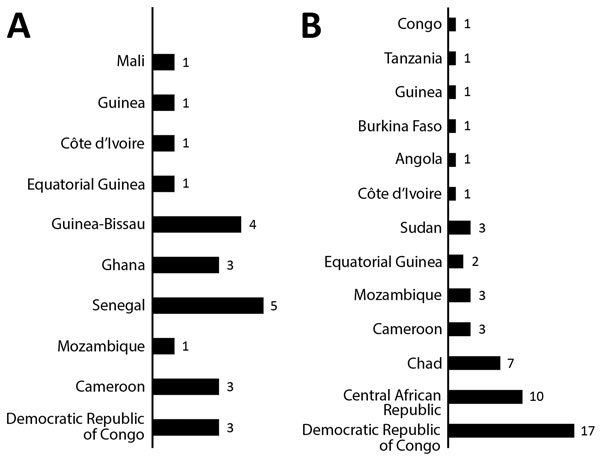
Countries of origin of patients infected with *Mansonella perstans* nematodes, Italy. A) Immigrants; B) expatriates. Immigrants were persons who were born in disease-endemic areas and then settled in Italy. Expatriates were persons from Italy residing in disease-endemic areas.

We identified countries in which *M. perstans* infections were acquired (Figure 2), and clinical and laboratory characteristics of the 74 patients (Table 1) and characteristics of patients who were infected only with *M. perstans* nematodes (33/74, 44.6%) (Table 2). However, we could not exclude other possible co-infections on the basis of screening tests performed (e.g., 23/33 patients came from country to which *Loa loa*, another filarial nematode, was endemic, and amicrofilaremic infections cannot be ruled out).

**Table 1:** Characteristics of 74 patients infected with *Mansonella perstans* nematodes, Italy*

Characteristic	Value
Age, y	48.9 (34.0−60.6)
Sex	
M	52 (70.3)
F	22 (29.7)
Viral co-infections	
HIV	4 (5.4)
HBV	3 (4.0)
HCV	3 (4.0)
HAV	1 (1.3)
Parasite diseases	
*Plasmodium falciparum* malaria	9 (12.1)
Giardiasis	2 (2.7)
Scabies	1 (1.3)
Other helminthiases	
Schistosomiasis	27 (36.4)
Strongyloidiasis	11 (14.9)
Hookworm infection	7 (9.4)
Loiasis	4 (5.4)
Trichuriasis	4 (5.4)
Onchocerciasis	2 (2.7)
Eosinophils/mm^3^	820 (470–1,270)
Eosinophil count >1,000/µL	32/73 (43.8)
Microfilaria/mL	62 (14–255)
Signs/symptoms	66 (89.2)
Abdominal pain	17 (23.0)
Arthralgia	10 (13.5)
Headache	11 (15.0)
Itching	25 (33.8)
Myalgia	2 (3.0)
Edema	11 (14.9)
Skin eruption	9 (12.2)
IgE >100 IU/mL	60/72 (83.3)
Antifilarial ELISA	49/53 (92.4)

*Values are median (IQR), no. (%), or no. positive/no. tested (%). HAV, hepatitis A virus; HBV, hepatitis B virus; HCV, hepatitis C virus; IQR, interquartile range.

**Table 2:** Characteristics of 33 patients infected only with *Mansonella perstans* nematodes, Italy*

Characteristic	Value
Eosinophils/mm^3^	620 (415–1,210)
Microfilaria/mL	32 (9.5–112)
Signs/symptoms	30 (90.9)
Abdominal pain	8 (24.2)
Arthralgia	5 (15.1)
Headache	5 (15.1)
Itching	12 (36.3)
Edema	6 (18.2)
Skin eruption	3 (9.1)

*Values are median (IQR) or no. (%). IQR, interquartile range.

Data for treatment were available for 60 (81.1%) of 74 patients. Most (34/60, 56.6%) patients were treated with levamisole (150 mg in 3 doses given every 48 h), followed by mebendazole (500 mg 3×/d for 15 d). After 2004, levamisole was no longer available, and patients were then treated with other drugs alone or in combination (doxycycline, mebendazole, ivermectin, diethylcarbamazine, albendazole, thiabendazole). Since 2009, first-line treatment has been mebendazole (500 mg 3×/d for 15 d), followed by doxycycline (100 mg 2×/d for 6 wks); this regimen was used for 11 (18.3%) of 60 patients. Clinical outcomes were available for only 5 of those patients, who showed complete clinical responses to the first-line treatment.

## Conclusions

Our series of 74 patients is one focused on imported infections with *M. perstans* nematodes. Identification of these infections is often complicated by co-infection with other infective agents. Bassene et al. analyzed patients infected only with *M. perstans* nematodes and concluded that these infections had little pathogenicity because infected persons were usually asymptomatic (*5*). Therefore, we considered as relevant identification of patients for whom other infections were excluded.

Our findings for this subgroup of patients are similar to those reported by Adolph et al. (*6*); however, we did not observe any major neurologic or psychological symptoms or extreme exhaustion. Among symptoms that we observed, transient swellings deserve particular attention. These swellings are similar to Calabar swellings caused by *L. loa* nematodes. When *L. loa* nematode infections are ruled out on the basis of an epidemiologic criterion (loiasis is present in a limited area of sub-Saharan Africa), *M. perstans* nematodes should be considered the probable cause of these swellings.

The proportion of patients with different grades of eosinophilia in our study is similar to that reported by Wiseman (*7*): ≈70% of those patients had >500 eosinophils/µL, and 45% had marked eosinophilia (>1,000 eosinophils/µL). Wiseman postulated that the proportion of symptomatic patients increased with increased eosinophil count (*7*). Conversely, increased eosinophil counts could be partially responsible for some symptoms, as reported by Fux et al. (*8*).

Of the 74 patients in our study, 68 (92.0%) had positive serologic results. One major strength of our study was that serologic analysis could be used to screen patients reporting compatible symptoms/signs and epidemiologic criteria. Thus, microscopic detection of microfilaremia, which requires more equipment and skills, could be used only for patients with positive serologic results.

Human infection with *M. perstans* nematodes raises questions about treatment (*1*) because of poor responses to standard antifilarial drugs and limited findings from controlled trials. In our case series, the first-line treatment changed over time on the basis of new evidence and availability of drugs. Therefore, we first administered mebendazole and levamisole on the basis of reports by Maertens and Wéry (*9*) and Wahlgren and Frolov (*10*). Subsequently, we administered mebendazole in combination with other drugs, as suggested by Bregani et al. (*11*). Since 2009, we have administered mebendazole plus doxycycline, according to the only available randomized clinical trial (*4*).

Our study had other limitations, which were caused mostly by the retrospective design. First, posttreatment follow-up was available for only a few patients because most resided only temporarily in Italy. Thus, we could not properly describe response to treatment. Second, most patients came to our center because of symptoms or an increased eosinophil count. Thus, the proportion of symptomatic patients is not representative of the general population with *M. perstans* nematode infections.

In summary, infection with *M. perstans* nematodes should be included in the differential diagnosis of patients with eosinophilia who have lived in disease-endemic countries. Serologic analysis (ELISA for filariae) can be used for screening, and detection of microfilaremia in peripheral blood should be performed for patients with positive serologic results.
